# Anxiety and Gene Expression Enhancement in Mice Exposed to Glyphosate-Based Herbicide

**DOI:** 10.3390/toxics10050226

**Published:** 2022-04-29

**Authors:** Yassine Ait bali, Nour-eddine Kaikai, Saadia Ba-M’hamed, Marco Sassoè-Pognetto, Maurizio Giustetto, Mohamed Bennis

**Affiliations:** 1 Department of Biology, Higher Normal School, University Mohamed V, Rabat 5118, Morocco; yassine.aitbali@gmail.com; 2 Laboratory of Pharmacology, Neurobiology, Anthropology, and Environment, Faculty of Sciences, Cadi Ayyad University, Marrakech 40000, Morocco; kaikai.noureddine@gmail.com (N.-e.K.); bamhamed@uca.ac.ma (S.B.-M.); mbennis@uca.ac.ma (M.B.); 3 “Rita Levi-Montalcini” Department of Neuroscience, University of Turin, 10124 Turin, Italy; marco.sassoe@unito.it

**Keywords:** glyphosate, anxiety, neuronal activation, c-Fos, pCREB

## Abstract

Growing evidence demonstrates that serotonin (5-HT) depletion increases activity in the amygdala and medial prefrontal cortex (mPFC), ultimately leading to anxiety behavior. Previously, we showed that glyphosate-based herbicides (GBHs) increased anxiety levels and reduced the number of serotoninergic fibers within the mPFCs and amygdalas of exposed mice. However, the impact of this 5-HT depletion following GBH exposure on neuronal activity in these structures is still unknown. In this study, we investigated the effects of GBH on immediate early gene (IEG) activation within the mPFCs and amygdalas of treated mice from juvenile age to adulthood and its subsequent effects on anxiety levels. Mice were treated for subchronic (6 weeks) and chronic (12 weeks) periods with 250 or 500 mg/kg/day of GBH and subjected to behavioral testing using the open field and elevated plus maze paradigms. Then, we analyzed the expression levels of c-Fos and pCREB and established the molecular proxies of neuronal activation within the mPFC and the amygdala. Our data revealed that repeated exposure to GBH triggers anxiogenic behavior in exposed mice. Confocal microscopy investigations into the prelimbic/infralimbic regions of the mPFC and in basolateral/central nuclei of the amygdala disclosed that the behavioral alterations are paralleled by a robust increase in the density and labelling intensity of c-Fos- and pCREB-positive cells. Taken together, these data show that mice exposed to GBH display the hyperactivation of the mPFC–amygdala areas, suggesting that this is a potential mechanism underlying the anxiety-like phenotype.

## 1. Introduction

Glyphosate (*N*-phosphonomethyl-glycine) is an organophosphorus pesticide that is widely used as a broad-spectrum, non-selective herbicide as well as a crop desiccant [1]. In the last decade, glyphosate-based herbicides (GBH) have been subjected to several clinical and experimental investigations, most of which have demonstrated considerable neurotoxic consequences [2,3,4]. In this regard, we previously showed that oral exposure to GBH in adolescent mice induced anxiogenic traits [5]. In fact, emotional abnormalities such as anxiety-like behavior have been raised as one of the common neurotoxic effects following GBH exposure [6,7].

A wealth of evidence from neuropsychological, neuroanatomical, and functional imaging studies in humans and animals indicated that the medial prefrontal cortex (mPFC) is a key component of the cortico–limbic–striatal circuits that are thought to generate pathological emotional behavior and the accompanying physiological disturbances [8,9]. In addition to the mPFC, the amygdala is also a key structure in the forebrain that regulates behaviors associated with anxiety, and its dysfunction may cause neurological disorders [10]. Indeed, both preclinical and clinical evidence pointed to neuronal hyperactivity in the amygdala as a pivotal factor contributing to emotional disturbances, such as pathological fear, aggression, and anxiety [11,12]. Such neuronal hyperactivity can be mapped by studying the expression of the IEG c-Fos [13] and the phosphorylation of the cAMP-responsive element-binding protein (CREB), an important master regulator of activity-dependent genes including c-Fos [14]. On the other hand, it has been previously demonstrated that the neuronal hyperactivity underlying anxiety behavior is mediated by the systemic depletion of serotonin (5-HT). In line with this, our previous results showed that the increased anxiety levels following exposure to GHB observed in mice were accompanied by a decreased number of serotoninergic fibers in both the mPFC and the amygdala [5]. Supporting these results, Martínez et al. [15] demonstrated that the oral administration of GBH to rats reduced the 5-HT levels in different brain regions, including in the cortex. However, the impact of 5-HT depletion following GBH exposure on neuronal activity in these structures is still unknown. Based on these previous findings, we hypothesized that there is a relationship between the GBH-induced elevated anxiety levels, 5-HT depletion, and the increase in neuronal activity within the mPFC and amygdala. Thus, in the present study, we assessed both c-Fos and pCREB expression by immunofluorescence and confocal microscopy to map neuronal activity in the brain in search of the possible mechanisms underlying anxiety-like behavior in GBH-exposed mice.

## 2. Methods

### 2.1. Animals

Male Swiss mice (1-month-old) were obtained from the animal husbandry department of the Faculty of Sciences, Cadi Ayyad University, Marrakech, Morocco. The animals were housed in Plexiglas cages (30 cm × 15 cm × 12 cm) under standard temperature (22 ± 2 °C) and photoperiod 12 h/12 h conditions and had free access to food and water. All procedures were conducted in accordance with approved institutional protocols and within the provisions for animal care and use prescribed in the scientific procedures on living animals, European Council Directive: EU2010/63. All efforts were made to minimize any animal suffering. The study was approved by the Council Committee of Research Laboratories of the Faculty of Sciences, Cadi Ayyad University, Marrakech, Morocco.

### 2.2. Pesticide

Roundup herbicide (glyphosate concentration 360 g/L in the form of glyphosate isopropylamine salt 486 g/L) with the molecular formula C_6_H_17_N_2_O_5_P, a molecular weight of 228.183 g/mol, a melting point 200 °C, and a density 1.218 g/cm^3^ was used in its liquid commercial form and supplied by the Monsanto Company (St. Louis, MO, USA).

### 2.3. Doses and Protocol of Exposure

Healthy mice were equally distributed to three experimental groups (*n* = 18/group): acute (unique administration), subchronic (6 weeks), and chronic (12 weeks). Each group was exposed to GBH daily through oral force-feeding at doses of 0 (control group, *n* = 6 animals), 250, or 500 mg/kg (treated groups, *n* = 6 animals each). These doses were selected based on Roundup’s no-observed adverse effect level (NOAEL), which was initially determined to be 500 mg/kg/day through our toxicity study (data not shown).

### 2.4. Behavioral Tests

All animals were tested between 9:00 h and 12:00 h during the light cycle. Before behavioral testing, they were gently handled and individually familiarized with the testing room and the test arena for 5 min. To minimize subjectivity, all behaviors were recorded and analyzed using the Ethovision XT Noldus 8.5 video tracking program (Noldus Information Technology b.v., Wageningen, The Netherlands) connected to a video camera (JVC, Yokohama, Japan).

#### 2.4.1. Open Field (OF)

This test is commonly used to assess locomotor activity and emotional reactivity in rodents placed in novel environments [16]. The test was performed as described by [17]. Briefly, the mouse was placed in the apparatus and allowed to move freely for 20 min. The time spent in the center area was used as an index of anxiety behavior and was recorded.

#### 2.4.2. Elevated Plus Maze (EPM)

This test is used to assess anxiety-like behavior in rodents [18]. Animals were tested individually for 5 min, as previously described by [17]. The time spent in the open arms (OA) and closed arms as well as the number of entries to each arm were quantified, allowing us to evaluate the OA ratio (time in OA /total time in both arms) and anxiety index, which was expressed as: 1 − [([time in OA/total time in both arms] + [OA entries/total number of entries])/2].

### 2.5. IEG Immunofluorescence Detection

Upon the conclusion of behavioral testing, the control and treated mice were anesthetized with an intraperitoneal injection of urethane 40% (1 g/kg, from Sigma–Aldrich, Saint-Quentin-Fallavier, France) and were transcardially perfused with saline solution (0.9%) followed by ice-cold 4% formaldehyde in phosphate-buffered saline (PBS; 0.1 M). The brains were then removed, post-fixed in the same fixative for 12 h, and cryoprotected overnight in 30% sucrose. They were then cut into 30 μm coronal sections on a freezing cryostat (Leica Microsystems, Wetzlar, Germany) as in [19]. The sections containing the mPFC and the amygdala were used for c-Fos and pCREB immunofluorescence. The regions of interest were determined according to the stereotaxic atlas of Paxinos and Franklin [20]. Free-floating sections were kept in PBS solution containing 0.05% Triton X-100 and 10% normal donkey serum (NDS) for 1 h followed by overnight incubation at RT with anti-pCREB (Monoclonal; 1: 300; Santa Cruz, sc-81,486, Santa Cruz, CA, USA) and anti-c-Fos (rabbit polyclonal; 1:500; Santa Cruz, sc-52, Santa Cruz, CA, USA) antibodies diluted in PBS with 3% NDS and 0.05 Triton X-100. The following day, the sections were washed and incubated with Cy3-conjugated anti-mouse secondary antibodies (1:1000; Jackson ImmunoResearch, West Grove, PA, USA) for 1 h at room temperature. After several PBS rinses, the sections were mounted on gelatin-coated glass slides and covered with Dako fluorescence mounting medium (Dako Italia, Milan, Italy).

### 2.6. Image Analysis

The analysis of c-Fos and pCREB immunofluorescence was analyzed from at least 3 cryosections per animal (three mice per experimental group) on five serial optical sections (1 μm Z-step size) that were acquired from the mPFC (prelimbic cortex, PrLCx, and infralimbic cortex, ILCx) and the amygdala (basolateral nucleus, BLA and central nucleus, CeA) with a laser scanning confocal microscope (LSM5 Pascal; Zeiss, DE, Jena, Germany) using a 20× objective (1.4 numerical aperture), and the pinhole was set at 1 Airy unit. After the images were processed for background subtraction using Image-J software (National Institute of Health, Bethesda, MD, USA), the ROI Manager tool in Image-J was employed to quantify both the cell density and integral optical density of the immunosignal levels. All of the analyses were carried out by an operator who was blinded to the experimental groups.

### 2.7. Statistical Analysis

The behavioral data and immunofluorescence results were compared between different groups (treated and control). A statistical analysis of the different independent variables was performed using two-way ANOVA (GBH dose and treatment duration) followed by a Holm–Sidak’s post hoc test for multiple comparisons.

## 3. Results

### 3.1. GBH-Exposed Mice Exhibit Higher Anxiety-like Levels in OF and EPM Tests

**Figure 1:**
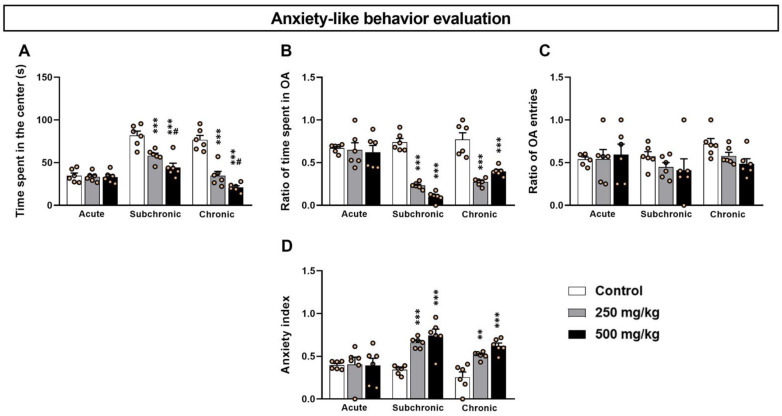
Effect of acute and repeated exposure to GBH on anxiety-like behavior. (**A**) Histograms showing time spent in the center of the OF, (**B**) the ratio of time spent in the open arms, (**C**) the ratio of open arm entries, and (**D**) the anxiety index. Results are presented as mean ± SEM. ** *p* < 0.01; *** *p* < 0.001; ^#^ *p* < 0.05. * indicates comparison between control vs. 250 mg/kg and 500 mg/kg groups; # indicates comparisons between 250 mg/kg vs. 500 mg/kg group.

In the OF test, the two-way ANOVA analysis of the time spent in the central area (Figure 1A) revealed significant differences among the treatment factors: dosage (F_(2.17)_ = 51.18, *p* < 0.001) and length (F_(2.17)_ = 38.04, *p* < 0.001), as well as the interaction between the two factors (F_(2.17)_ = 13.22, *p* < 0.001). The post hoc analysis confirmed that the subchronic and chronic treated groups (both 250 and 500 mg/kg) exhibited a significant decrease in the time spent in the central zone of the OF (*p* < 0.001) (Figure 1A).

Supporting the OF results, the analysis of the data derived from the EPM test (Figure 1B–D) revealed a significant difference in both the time spent in the OA and the anxiety index among the treatment dosage (F_(2.17)_ = 1.57, *p* < 0.001; F_(2.17)_ = 17.75, *p* < 0.001) and length (F_(2.17)_ = 24.35, *p* < 0.001; F_(2.17)_ = 8.77, *p* < 0.001) as well as the interaction of the two factors (F_(2.17)_ = 10.48, *p* < 0.001; F_(2.17)_ = 4.53, *p* < 0.001). In contrast, the number of open arm entries showed no significant differences for both the treatment (F_(2.17)_ = 1.57, *p* > 0.05) and length (F_(2.17)_ = 1.55, *p* > 0.05) (Figure 1C), and there was no significant interaction in the two factors (F_(2.17)_ = 0.83, *p* > 0.05). Finally, the post hoc comparisons confirmed that the sub-chronic and chronic treated groups showed a significant decrease in the time spent in the open arm (*p* < 0.001) and an increase in the anxiety index (*p* < 0.01) with respect to the controls (Figure 1B–D). However, no statistical differences were detected between the groups following acute exposure (*p* > 0.05).

### 3.2. GBH-Exposed Mice Display Increased IEG Expression in the mPFC and the Amygdala

**Figure 2:**
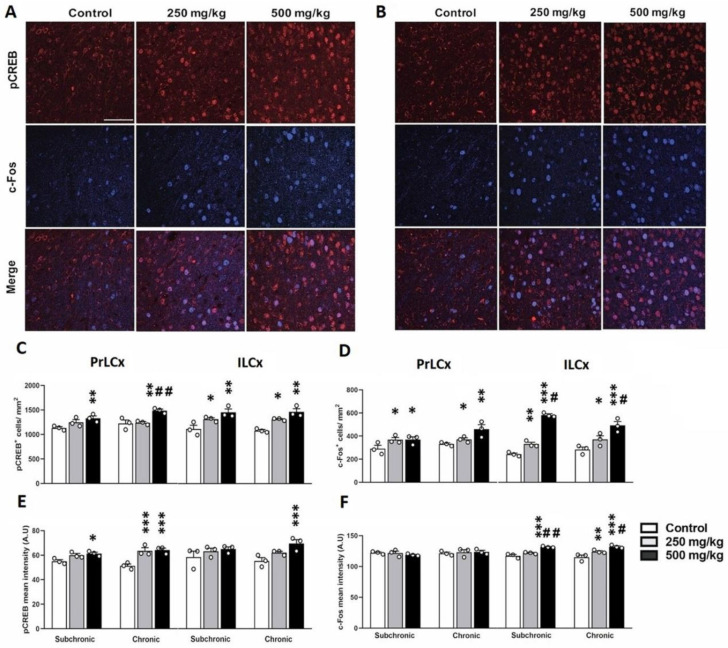
Effects of GBH on pCREB and c-Fos expression in the mPFC. Representative confocal images illustrating pCREB^+^ and c-Fos^+^ distribution in the PrLCx (**A**) and the ILCx (**B**) of animals injected either with GBH or vehicle for a chronic period. Histograms showing the analyses of pCREB^+^ (**C**) and c-Fos^+^ (**D**) cell density. Optical density analysis of pCREB (**E**) and c-Fos (**F**) immunofluorescence. * *p* < 0.01; ** *p* < 0.01; *** *p* < 0.001; # *p* < 0.05; ## *p* < 0.01. * indicates comparison between control vs. 250 mg/kg and 500 mg/kg groups; # indicates comparisons between 250 mg/kg vs. 500 mg/kg group. Scale bar: 10 μm.

Given that the abnormal neuronal activity within the mPFC and the amygdala correlates with impaired mood and anxiety disorders [11], we examined whether the GBH-exposed mice exhibited altered neuronal activation within these two brain regions in parallel with increased anxiety-like behavior. To achieve this, we investigated the cellular expression of both c-Fos and pCREB and established molecular markers of neuronal activity [21,22]. Our immunofluorescence data (Figure 2A,B) show that the GBH-exposed mice exhibited g levels of activation in the PrLCx and ILCx that were generally higher, as indicated by an increased number of pCREB^+^ and c-Fos^+^ cells and by an increased mean labeling intensity that varied between the areas that were analyzed and the differential treatments (Figure 2C–F, Table S1).

**Figure 3:**
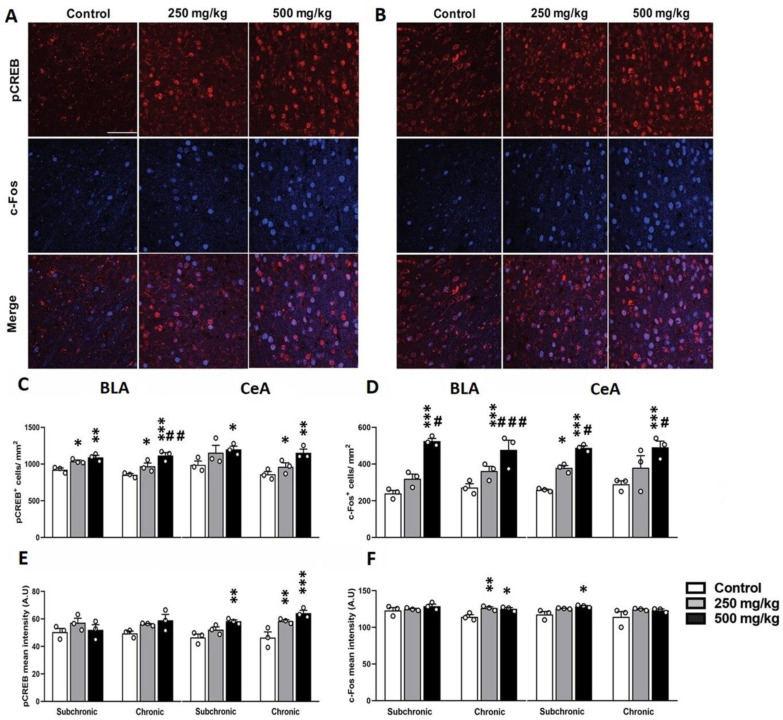
Effect of GBH on the pCREB and c-Fos expression in the amygdala. Representative confocal images illustrating pCREB^+^ and c-Fos^+^ distribution in the BLA (**A**) and the CeA (**B**) from animals who were chronically exposed. Cell density of pCREB (**C**) and c-Fos (**D**). Optical density of pCREB (**E**) and c-Fos (**F**). * *p* < 0.01; ** *p* < 0.01; *** *p* < 0.001. # *p* < 0.05; ## *p* < 0.01; ### *p* < 0.001. * indicates comparison between control vs. 250 mg/kg and 500 mg/kg groups; # indicates comparisons between 250 mg/kg vs. 500 mg/kg group. Scale bar: 10 μm.

Moreover, the general increased expression of c-Fos and pCREB immunofluorescence (Figure 3A,B) was also observed in the BLA and CeA in GBH-exposed mice (Figure 3C–F, Table S2). These results suggest that GBH intake can change gene expression levels in the neurons of the mPFC and the amygdala, representing the hyperactivation of these circuits.

## 4. Discussion

While recent studies have increased concerns about the potential neurotoxicity of glyphosate and GBH [7,23,24], data regarding their effects on anxiety mechanisms are partial and fragmented. In this study, we investigated IEG expression in the anxiety-related brain regions of mice treated with sub-toxic doses of GBH with the aim to assess the neuronal basis of the behavioural signs and to disentangle relevant molecular mechanisms.

We found that GBH-exposed mice exhibited increased anxiety-like behavior that is paralleled by the increased activation of both the mPFC and the amygdala structures, further corroborating previous reports on the crucial involvement of these brain structures in anxiety responses [25]. Previous studies highlighted that low levels of 5-HT can facilitate mPFC and amygdala neuronal hyperactivity, a hallmark characteristic of patients with emotional disorders [26]. Accordingly, the increased neuronal activity observed within the mPFC and the amygdala of GBH-exposed mice correlates with our previous observations on alterations to the 5-HT system. Indeed, the GBH-exposed mice show a decreased number of 5-HT^+^ neurons in the raphe nucleus and a lower density of 5-HT^+^ fibers in both the ILCx and BLA [5]. It has been well described that the 5-HT system has anatomical connections with the ventral mPFC. This region contains a high density of 5-HT transporter sites [27] and 5-HT receptors, including the 5-HT1A and 5-HT2A subtypes [28]. In line with this, the activation of the midbrain raphe nuclei evokes the inhibition of neurons in the ventral mPFC via the activation of the 5-HT1A receptors [29]. Similarly, 5-HT primarily plays an inhibitory role in amygdala output by both increasing local-circuit GABAergic inhibition and by decreasing glutamatergic excitatory output [30]. Therefore, 5-HT depletion can increase the excitability of these two structures (mPFC and amygdala) by removing these inhibitory components. Thus, our data, which indicate for the first time that the 5-HT system defects following GBH exposure facilitate abnormal emotional behavior through the induction of neuronal hyperactivity in the mPFC and amygdala, are consistent with previous studies. Moreover, our findings are also consistent with fMRI data showing that in patients who have been diagnosed with mood disorders, there is an excessive amygdala response (especially CeA) as a possible result of PFC–amygdala loop modification [31].

Transcription factors, such as c-Fos and pCREB, are upregulated by dynamic changes in electrical activity within a cell and function to cause changes in gene transcription within the same cell [32]. While the pCREB/CREB ratio is a valuable approach to study the expression level of the total CREB and subsequently the level of its phosphorylated form [33,34], several reports have used immunofluorescence pCREB as an indicator of neuronal activity [35,36]. pCREB expression most commonly reflects the activation of the second messenger cAMP [37] or glutamate binding to the neuronal NMDA glutamatergic receptors (NMDAR), causing subsequent increases in intracellular Ca^2+^ [38]. Thus, in view of our previous observations, the increased expression of pCREB that we observed after GBH exposure is likely produced by Ca^2+^ influx through the elevated NMDAR activation that is produced by either the increased expression of the NMDA receptors [21] or, alternatively, by the excessive release of glutamate by activated astrocytes [2]. Whether the observed increase in pCREB levels produced by GBH could be attributable to the concomitant increase in the expression of total CREB remains to be clarified. In addition, the GBH-dependent hyperactivation of NMDAR could also recruit other signal transduction pathways through the activation of protein kinases such as the Ca^2+^/calmodulin-dependent protein kinase II and the extracellular signal-regulated kinase [2]. This hyperactivation can also explain the upregulation of c-Fos expression that can be further reinforced by pCREB binding to the promoter region of c-Fos [39]. Taken together, our data suggest that the GBH-induced high influx of Ca^2+^ through the NMDARs could explain the aberrant neuronal activity resulting in increased pCREB and c-Fos levels.

Alternatively, previous studies have suggested other mechanisms whereby the dysfunction of the 5-HT system following GBH exposure can increase activity in the amygdala. Indeed, in different areas of the central nervous system, the 5-HT1A receptor subtype mediates postsynaptic inhibition [40]. However, the low levels of 5-HT1A receptor expression observed in the BLA suggest that the 5-HT-mediated inhibition of BLA cell firing does not result from the direct activation of postsynaptic 5-HT1A receptors but rather by the inhibition of glutamate release via the activation of presynaptic 5-HT1A receptors [12]. Moreover, the expression of the 5-HT3 and 5-HT2 receptor subtypes on the interneurons of the BLA suggest that the inhibition of cell firing may also be facilitated by the direct excitation of GABAergic interneurons in this nucleus [41]. Thus, we suggest that the increased activity within the BLA of GBH-treated mice likely involves not only enhanced NMDAR activity, but also the lower activation of GABAergic interneuron in view of the 5-HT depletion that we observed in the BLA following GBH exposure. Importantly, further studies involving electrophysiological recording from different neuronal populations (excitatory and inhibitory cells) after GBH exposure will be necessary to further shed light on these mechanisms.

Overall, our data identified the putative cellular mechanisms of the anxiogenic effect of GBH exposure, thus increasing our understanding regarding the neurotoxicity of this pesticide. We also provide evidence that bridges the relationship between GBH exposure, 5-HT defects, and neuronal hyperactivity in regulating emotional behavior.

## Data Availability

Not applicable.

## Supplementary Materials

The following are available online at https://www.mdpi.com/article/10.3390/toxics10050226/s1, Table S1: Effects of GBH on the mPFC activity, Table S2: Effects of GBH on amygdala activity.

## Abbreviations

5-HT	Serotonin
BLA	Basolateral amygdala
CeA	Central amygdala
EPM	Elevated plus maze
GBH	Glyphosate-based herbicide
IEGs	Immediate early genes
IL	Infralimbic cortex
mPFC	medial prefrontal cortex
NOAEL	No-observed adverse effect level
NDS	Normal donkey serum
OA	Open arm
OF	Open field
PBS	Phosphate-buffered saline
pCREB	cAMP-responsive element-binding protein
PrLCx	Prelimbic cortex
