# Supplementary material for: Anxiety and Gene Expression Enhancement in Mice Exposed to Glyphosate-Based Herbicide
Source: Toxics. 2022 Apr 29;10(5):226. doi: 10.3390/toxics10050226 (PMC9147174; doi:10.3390/toxics10050226)
Supplement: Supplementary file 1 [file toxics-10-00226-s001.zip › toxics-1684467-supplementary.pdf]

toxics

Supplementary Materials: Anxiety and Gene Expression Enhancement in Mice Exposed to Glyphosate-Based Herbicide

Yassine Ait bali, Nour-eddine Kaikai, Saadia Ba-M’hamed, Marco Sassoè-Pognetto, Maurizio Giustetto and Mohamed Bennis

Table S1. Effect of GBH on the mPFC activity.

		Control	250 mg/kg	500 mg/kg	ANOVA					
					Treatment		Duration		Treatment x Duration	
pCREB	**PrLCx**				*F*	*p*	*F*	*p*	*F*	*p*
	Cell density									
	Subchronic	1132.96 ± 25.47	1249.19 ± 53.07	1332.96 ± 45.38 **	14.65	***	4.84	ns	1.77	ns
	Chronic	1225.10 ± 68.46	1239.76 ± 22.38	14,487.93 ± 30.68 **,##						
	Optical density									
	Subchronic	55.11 ± 1.26	60.02 ± 1.32	61.34 ± 1.18 *	21.26	***	0.44	ns	3.20	ns
	Chronic	51.29 ± 1.54	63.64 ± 2.43 ***	64.20 ± 1.61 ***						
	ILCx									
	Cell density									
	Subchronic	1113.06 ± 76.36	1314.11 ± 28.17*	1454.42 ± 66.19 ***	25.36	***	0.4	ns	0.08	ns
	Chronic	1080.60 ± 21.98	1312.01 ± 13.61 **	1463.84 ± 63.24 ***						
	Optical density									
	Subchronic	58.50 ± 4.89	63.18 ± 2.53	65.06 ± 1.77	6.22	*	0.01	ns	0.9	ns
	Chronic	55.34 ± 2.73	62.12 ± 1.11	69.56 ± 3.16 **,##						
c-FOS	PrLCx									
	Cell density									
	Subchronic	291.09 ± 29.31	368.57 ± 19.97 *	369.62 ± 23.10 *	8.97	**	4.81	*	1.61	ns
	Chronic	330.88 ± 9.12	370.67 ± 14.50	459.67 ± 39.08 **,#						
	Optical density									
	Subchronic	122.32 ± 2.33	121.82 ± 3.26	118.93 ± 0.97	0.09	ns	0.74	ns	0.63	ns
	Chronic	121.47 ± 2.01	123.22 ± 4.10	123.86 ± 2.41						
	ILCx									
	Cell density									
	Subchronic	242.92 ± 9.98	330.88 ± 15.21 *	582.18 ± 10.62 ***,###	76.84	***	0.02	ns	5.46	ns
	Chronic	283.76 ± 21.63	370.67 ± 31.77 *	492.13 ± 33.65 ***,##						
	Optical density									
	Subchronic	117.49 ± 2.05	121.94 ± 0.87	131.74 ± 0.73 ***,##	34.51	***	0.07	ns	0.93	ns
	Chronic	115.21 ± 3.40	124.19 ± 1.43 **	132.19 ± 1.47 ***,#						

PrLCx: Prelimbic cortex, ILCx: Infralimbic cortex; * $p < 0.05$; ** $p < 0.01$; *** $p < 0.001$; ns (no significant) $p > 0.05$; # $p < 0.05$; ## $p < 0.01$; ### $p < 0.001$. * indicates comparison between control vs. 250 mg/kg and 500 mg/kg groups; # indicates comparisons between 250 mg/kg vs. 500 mg/kg group.

Table S2. Effect of GBH on the amygdala activity.

		Control	250 mg/kg	500 mg/kg	ANOVA					
					Treatment		Duration		Treatment x duration	
	**BLA**				*F*	*p*	*F*	*p*	*F*	*p*
	**Cell density**									
	**Subchronic**	922.49 ± 23.32	1037.67 ± 16.85*	1090.03 ± 29.18**	24.98	***	2.4	ns	1.63	ns
	**Chronic**	850.24 ± 17.61	968.56 ± 45.02 *	1115.16 ± 40.10 ***,##						
	**Optical density**									
	**Subchronic**	50.42 ± 2.67	57.26 ± 3.26	52.00 ± 3.83	2.89	ns	0.46	ns	1.18	ns
	**Chronic**	49.49 ± 1.64	56.19 ± 0.57	59.01 ± 4.30						
**pCREB**	**CeA**									
	**Cell density**									
	**Subchronic**	988.46 ± 52.57	1153.90 ± 100.49	1199.97 ± 42.88 *	8.93	**	6.33	*	0.73	ns
	**Chronic**	858.62 ± 38.44	962.28 ± 52.67	1152.85 ± 50.26 **,#						
	**Optical density**									
	**Subchronic**	46.58 ± 2.46	52.20 ± 1.96	58.28 ± 1.09 **	20.03	***	4.45	ns	1.18	ns
	**Chronic**	46.46 ± 4.11	58.49 ± 0.94 **	64.23 ± 1.99 ***						
	**BLA**									
	**Cell density**									
	**Subchronic**	240.83 ± 15.84	320.41 ± 24.39	524.59 ± 14.84 ***,###	37.62	***	0.12	ns	1.42	ns
	**Chronic**	272.24 ± 21.02	361.25 ± 26.15 *	477.47 ± 52.21 ***,#						
	**Optical density**									
	**Subchronic**	123.07 ± 3.90	124.88 ± 1.02	128.96 ± 2.77	6.28	*	3.17	ns	1.99	ns
**c-FOS**	**Chronic**	114.49 ± 2.78	126.27 ± 1.67 **	125.23 ± 1.70 *						
	**CeA**									
	**Cell density**									
	**Subchronic**	259.68 ± 4.56	378.00 ± 13.85 *	487.94 ± 10.93 ***,#	22.17	***	0.19	ns	0.12	ns
	**Chronic**	290.04 ± 19.39	379.05 ± 66.39	491.09 ± 33.45***,#						
	**Optical density**									
	**Subchronic**	117.64 ± 3.85	125.80 ± 0.40	128.81 ± 0.99 *	5.32	*	1.32	ns	0.18	ns
	**Chronic**	114.41 ± 7.17	124.67 ± 0.81	123.49 ± 1.57						

BLA: Basolateral amygdala, CeA: Central Amygdala; * $p < 0.05$; ** $p < 0.01$; *** $p < 0.001$; ns (no significant) $p > 0.05$; # $p < 0.05$; ## $p < 0.01$; ### $p < 0.001$. * indicates comparison between control vs. 250 mg/kg and 500 mg/kg groups; # indicates comparisons between 250 mg/kg vs. 500 mg/kg group.
